# Clinical Research in Resource-Limited Settings: Enhancing Research Capacity and Working Together to Make Trials Less Complicated

**DOI:** 10.1371/journal.pntd.0000619

**Published:** 2010-06-29

**Authors:** Trudie A. Lang, Nicholas J. White, Tran Tinh Hien, Jeremy J. Farrar, Nicholas P. J. Day, Raymond Fitzpatrick, Brian J. Angus, Emmanuelle Denis, Laura Merson, Phaik Yeong Cheah, Roma Chilengi, Robert Kimutai, Kevin Marsh

**Affiliations:** 1 Centre for Vaccinology and Tropical Medicine, University of Oxford, Churchill Hospital, Oxford, United Kingdom; 2 KEMRI-Wellcome Trust Research Programme, Kilifi District Hospital, Kilifi, Kenya; 3 Mahidol Oxford Research Unit, Bangkok, Thailand; 4 Hospital for Tropical Diseases, District 5, Ho Chi Minh City, Vietnam; 5 Oxford University Clinical Research Unit, District 5, Ho Chi Minh City, Vietnam; 6 Department of Public Health, University of Oxford, Old Road Campus, Oxford, United Kingdom; Case Western Reserve University School of Medicine, United States of America

Clinical trials conducted in developing countries differ in many respects to those carried out in the West; for example, they are usually conducted in vulnerable populations, focus mainly on infectious diseases, and often have severe endpoints. In these regions, trial capacity lags behind that of wealthier nations, particularly in terms of the ability of research sites to lead broad and independent clinical research programmes. Product development trials are important for the registration of new treatments and vaccines, yet do not leave sites with the skills to run their own trials, as protocol design, operational planning, and data management are typically conducted remotely by the sponsor. There is also a need for more disease management trials to examine and then improve health outcomes, but the capacity to design and execute such studies is often absent. The process of increasing clinical trial capacity should be led by the research sites and tailored to their needs, as trial methods and guidelines need to be appropriately designed and crafted to be fit for purpose in the developing country context. We discuss the need to address the deficit in capacity and training and propose a collaborative solution for identifying the gaps and then designing methods, guidance, and sharing approaches to make clinical trials less daunting and cumbersome, particularly when being planned for resource-limited settings.

## Trials in Resource-Limited Settings

Clinical trials establish the evidence base for the prevention and treatment of disease. They are critically important in developing countries, not simply because this is where the potential is greatest for improving health in numerical terms (as these regions have the highest diseases burden), but also because there is enormous potential gain from effective new interventions and because these populations have been under-represented in clinical research to date. The human and material resource capacity available to ensure a high standard of design, management, and operation of clinical trials in developing countries lags far behind that available in wealthier nations.

**Figure 1:**
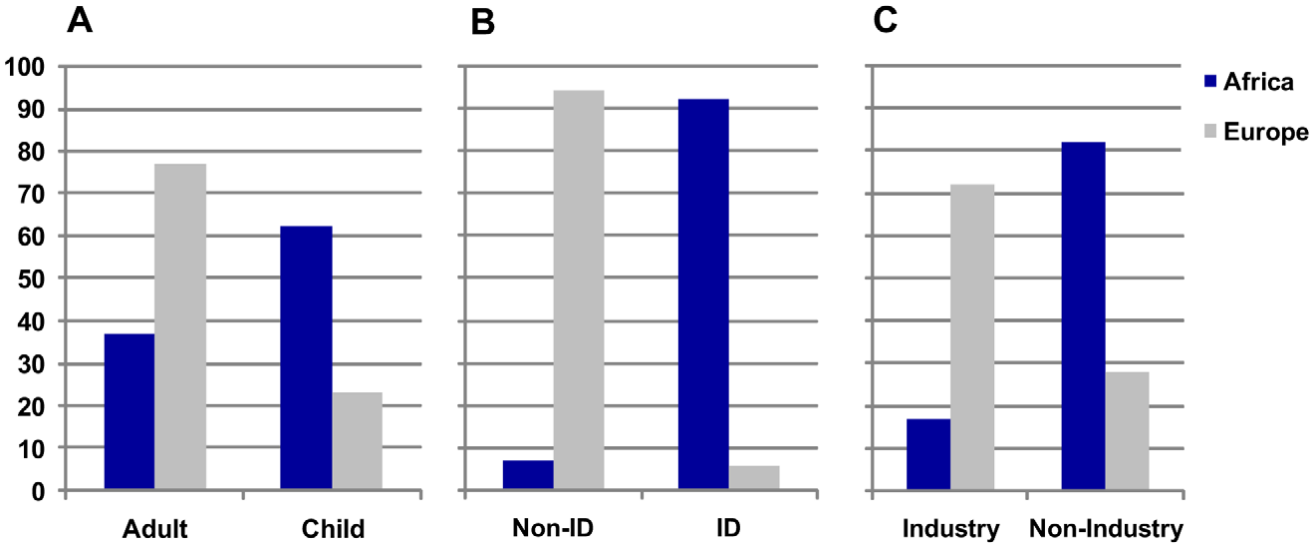
Trials differ in Europe and Africa. Classification of a random sample of 100 trials for each of five countries in Europe (France, Germany, Italy, Spain, and the United Kingdom) and Sub-Saharan Africa (Kenya, Mali, Tanzania, Uganda, and Zambia). Trials in Africa focus predominantly on paediatric populations (A) and infectious disease (B) and are non-industry sponsored (C). Data were abstracted from the ClinicalTrials.gov website in August 2009.

Although many of the issues confronting clinical trialists working in resource-limited settings are the same as those affecting academic researchers in the wealthier regions of the world, there are significant differences which both highlight the issues involved and require specific attention. In contrast to clinical trials in wealthy countries, those in developing countries frequently have endpoints that are severe disease outcomes or mortality. They more often involve children, focus predominantly on infectious diseases, and are more often sponsored by not-for-profit organisations. We illustrate this difference by taking a random sample of 100 trials registered at ClinicalTrials.gov for each of the top five countries in Europe and Africa and then classifying them (Figure 1). Public health problems that are specific to developing countries also urgently require more and better clinical trials to inform policy, such as the management of disease outbreaks (including those with pandemic potential) in displaced populations, in refugee camps, and following natural disasters. These very specialised situations and environments need new, highly practical, and appropriate interpretation of regulations and guidelines to enable rapid and flexible trial implementation.

## All Those Guidelines and Regulations

Over recent years there has been massive proliferation of regulations affecting the conduct of clinical trials. This process began in 1964 with the Declaration of Helsinki made by the World Medical Association (WMA) in response to a tightening of legislation following the thalidomide disaster in the 1960s [1], [2]. The implementation of the declaration resulted in the US Food and Drug Administration (FDA) having to reject trial data from countries with ethical and safety standards that differed from the US. The perceived differences between standards drove the harmonisation process led by regulators from Japan, Europe, and the US, and experts from the pharmaceutical industry, who produced, in 1996, the International Conference on Harmonisation of Technical Requirements for Registration of Pharmaceuticals for Human Use - *Good Clinical Practice* (ICH-GCP) guidelines [3].

Pre-dating ICH-GCP, the Council for International Organizations of Medical Sciences (CIOMS) produced its *International Ethical Guidelines for Biomedical Research Involving Human Subjects* in 1982 [4]. Revised in 2002, these guidelines are intended to guide lower-income countries in applying the ethical principles that were laid out in the Declaration of Helsinki. Another set of international clinical trial guidelines was produced by the World Health Organization in 1995 [5]. The WHO *Guidelines for good clinical practice (GCP) for trials on pharmaceutical products* were developed to provide a global standard for clinical trials. They were intended to complement existing regulations in those WHO member states that had already enforced clinical trials legislation or to provide a basis for new regulations in countries that had not.

However, neither the CIOMS nor the WHO guidelines hold the force of law, and ICH-GCP is now the d*e* facto global standard by which trials are run and has become a legal requirement for clinical trial conduct in many countries. Consistent criticisms of ICH-GCP are that it is outdated, that not enough countries were involved in its development, and that it is focussed on the needs of industry and drug registration with minimal representation from academia and noncommercial organisations [6], [7], [8], [9]. It is also focussed on drugs as the intervention and so is difficult to apply to other trials and broader types of clinical research that would benefit from sensible and appropriate quality and ethical guidance.

The Declaration of Helsinki can also be difficult to apply. For example, the position of the WMA to insist that a medical doctor be responsible for taking informed consent is not practical or always appropriate. In our research sites, community-based trials are very important to assess proposed improved or new interventions. Here, where there are rarely doctors present, it would be inappropriate to introduce a doctor where normally there are nurses or clinical officers just for the purposes of the trial. In these situations it could be argued that the profession responsible for administration of the intervention is much better placed to request fully informed consent from potential participants.

In the US and Europe, as the regulations and guidelines have become more strongly enforced and embedded in legislation, it has been increasingly recognised that a high level of support is necessary to help researchers run their trials. Recently a network has been set up in the UK specifically to support clinical trials in children. The Medicines for Children Research Network [10] is funded by the UK's Department of Health and recognises that conducting trials in children has very specific challenges and needs dedicated experts to provide tools and guidance. After the European Union made ICH-GCP a legal requirement in 2004, the UK's Medical Research Council (MRC) launched a Web site to help noncommercial trialists find their way through the guidelines and direct them in what they need to ensure that their trials are legal and compliant [11]. Since 2004, most UK universities now have clinical trials offices that support their academics in conducting clinical trials. Universities have made these provisions because as sponsors they bear the legal burden of ensuring that trials do not breach ICH-GCP.

In the US, the FDA has partnered with Duke University to establish the Clinical Trial Transformation Initiative [12]. Their aim is to generate evidence on the conduct of clinical trials that will improve their quality and efficiency. This is a US-focussed exercise that will examine current practice under FDA requirements and make recommendations to improve trial conduct in the US so it is more straightforward and attractive to researchers.

These various initiatives set out to unravel the guidelines and facilitate trial conduct for non-commercial researchers in their distinct environments. The same is needed for the unique setting of developing country-based trials. However, any such initiative should be led from the perspective of these regions and by the researchers working there. It should be highly collaborative and must reflect the real issues and gaps, which will need to be continuously captured and monitored. In addition, it must be broad enough to provide support for all areas of trial conduct, from governance and insurance issues through to trial design and operations, and also have a strong focus on training and career development. Any guidelines or recommendations put forward must be derived through a participatory action research process to ensure that they address the gaps and are appropriate and practicable for this challenging environment.

## Trials in Resource Limited Settings, the Current Situation

The majority of clinical trials conducted in developing countries have sponsors who are based in Europe or the US. Trial sponsors frequently demand that research sites implement the sponsor's own interpretation of ICH-GCP, which is often over and above what is actually required. This is understandable as ICH-GCP is generally seen as the ‘gold standard’ for all clinical research and the investigational product research is typically aimed at US FDA or European Medicines Agency (EMA) licensure [9], [13]. However, these exacting standards and the associated burden of process and paperwork can be daunting for academic researchers and are frequently inappropriate where they work [8]. A more locally appropriate interpretation of GCP guidelines is often possible and provides just as high a standard in terms of ethics and quality, but investigators either lack the confidence to develop and propose pragmatic alternatives or are not aware that they can and should.

As well as product development studies there is also a need for more disease management trials. These are often large yet straightforward trials that assess whether new approaches could be made in current treatment or care practices to improve outcomes. Typically these trials assess known drugs that have been widely used in other settings. These studies can make a significant impact on public health practice. Examples of potential trial topics include managing malnutrition or prescribing antibiotics during childbirth. New or adapted interventions can potentially bring about dramatic reductions in mortality, but they must be supported by sound medical evidence, and researchers need access to tools and training in order to undertake trials to obtain this evidence. Disease management studies such as these that assess new uses for existing or licensed practices or treatments are normally associated with lower risk than trials that evaluate new treatments. While the basic principles of GCP are straightforward and their application is important to ensure high standards in ethics and data quality, little guidance exists on how they should be interpreted and applied in disease management or other non-investigational new product trials. It is important to remember that these are *guidelines*, intended to be subject to varying interpretation and application. Within ICH-GCP itself it is repeatedly stated that the guidelines should be interpreted and applied in a manner appropriate to the risk of the research. Pragmatic interpretation is needed, because many aspects of ICH-GCP are not applicable in disease management trials and can be problematic when applied to investigational new product trials, especially in the specific situations found in resource-limited settings. Additionally, there are fundamental areas, such as randomisation, that ICH-GCP does not cover. Robust randomisation of subjects into groups is critical to rigorous trial design, and its reliable implementation is fundamental to producing a valid dataset—i.e., the trial giving the right answer! Straightforward guidance is required to ensure that this can been done securely and accurately.

## The Need for a Research-Led, Developing Country–Specific Clinical Trial Programme

A major criticism of ICH-GCP is that it was developed through a process of informal consensus rather than through research or evidence of best practices [6]. While programmes like the Duke-FDA Clinical Trials Transformation Initiative are seeking to gather evidence to improve trial conduct in the US, no such initiatives exist to support a broad range of trials in the developing world.

A collaborative programme that is designed specifically to support developing country-based trials and is not disease specific could benefit researchers in developing countries. It would work best if participation were free and available online. Such a collaboration could encourage disease management research and product development trials, and could give researchers access to all they need from training right through to template documents, suggested operating procedures, and guidelines, all accessed from one site. An important element of such a resource is that it could offer advice, tools, and guidance appropriately adjusted for all types of trials with varying levels of risk. As it would be specifically for researchers working in these settings, it would be able to focus on relevant issues such as community participation in trials, which is of particular importance when trials are being conducted in vulnerable populations. Ultimately it could provide sensible and pragmatic interpretations of good clinical practice guidelines derived using an evidence-based approach and make the conduct of high-quality trial conduct easier, less cumbersome, and much less daunting. Such a site would benefit from being highly interactive and allow researchers to share their tools, experiences and interpretations of the guidelines.

It is important to emphasise that the need to facilitate trial conduct in resource-limited settings does not mean developing an approach that is in any sense substandard or inferior. Developing country trials require at least the same degree of attention to the quality of processes and procedures and of data management as that required in resource-rich settings—and the attention to international ethical standards may need to be even greater where vulnerable populations are involved. A focussed effort is needed to establish a straightforward system by which researchers can navigate the regulations and guidelines and determine what is needed for their planned research and appropriate for the context in which it will be carried out.

## Training and Professional Development

In Europe and the US being a Clinical Trials Scientist or ”trialist” is a well recognised profession. There are well established and recognised professional bodies [14], [15], and numerous vocational and academic qualifications are available from diplomas through to doctorates, all specific to the science and profession of designing and conducting clinical trials. Clinical trialists of all disciplines can be found in universities, health organisations, medical research charities, and industry.

In the regions where we work there is limited recognition of the clinical trialist as a profession or viable career path. We believe this is a key factor impeding capacity development. Many still see the running of trials as an administrative function. Taking a clinical question and then developing a protocol and conducting a clinical trial to answer that question is a research discipline that requires training, experience, and critical thinking. Only when there is a critical mass of skilled trial coordinators, laboratory technicians, data managers, statisticians, monitors, research nurses, and investigators will research sites be in a position to design and lead their own programmes.

Clinical trial staff in these regions would benefit from a free online continuing professional development scheme that allows them to register their role, experience, training, and core competencies, and then build points to track their competencies and new training. This scheme should be linked to e-learning opportunities and to a local network of shared real-life training opportunities and mentoring.

## Summary

Our aim is to raise awareness of the issues faced by researchers in developing countries and to introduce an initiative we are developing.

We propose that the gaps and issues we have outlined could be largely addressed by building a community of researchers from all the various roles who will be able to access the information, guidance and resources they need, whilst also be able to share methods and pragmatic operational practices that have been locally derived and known to work. Some examples include template consent forms, data management systems, and example protocols and laboratory sample collection and handling methods.

We emphasize that this initiative is entirely based on an ethos of collaboration, open access, and sharing practice; indeed it will only be successful if research groups both use the resource and contribute to its development. The development of a prototype of web site for this initiative is underway and can be found at http://pilot.globalhealthtrials.org/. We are making this public at this early juncture as we are seeking involvement from our colleagues right from the outset in line with the open and collaborative ethos that is envisaged. Therefore, we encourage colleagues to become part of this initiative by providing content, commenting on the Web site, and sharing their operational tools. We also welcome all those engaged in trials to register and build their own personal professional development record to track their career and training record, and to provide a review structure.

## Conclusion

To improve clinical trial conduct in resource-limited settings we need easier operational tools and guidance as well as skilled staff. This needs to be more than conducting externally sponsored trials that are designed and led elsewhere. We suggest true capacity-building might be best achieved by establishing a community of developing country based researchers to share locally derived solutions and build a set of validated methods and operational tools that will enable pragmatic and locally led development.
